# Neovascular Glaucoma as the First Symptom of Bilateral Occlusive Retinal Vasculitis in a 4-Year-Old Girl: A Case Report

**DOI:** 10.3390/biomedicines13010148

**Published:** 2025-01-09

**Authors:** Klaudia Rakusiewicz-Krasnodębska, Joanna Jędrzejczak-Młodziejewska, Krystyna Kanigowska, Wojciech Hautz

**Affiliations:** Department of the Pediatric Ophthalmology, Children’s Memorial Health Institute, 04-730 Warsaw, Poland

**Keywords:** neovascular glaucoma, pediatric ophthalmology, occlusive retinal vasculitis (ORV), retinal ischemia, anti-vascular endothelial growth factor therapy

## Abstract

Neovascular glaucoma is a rare and serious condition typically associated with advanced ocular or systemic vascular diseases such as central retinal vein occlusion or diabetic retinopathy. This report describes a unique case of neovascular glaucoma presenting for the first time as an initial symptom of bilateral occlusive retinal vasculitis (ORV) in a generally healthy 4-year-old girl. The patient presented with symptoms of pain and redness in the left eye, accompanied by high intraocular pressure. These symptoms were particularly distressing and uncharacteristic for such a young child. Clinical examination revealed significant findings, including elevated intraocular pressure, corneal edema, and iris neovascularization in the left eye. Additional imaging studies, including fluorescein angiography, demonstrated extensive retinal ischemia with peripheral capillary nonperfusion, confirming the diagnosis of occlusive vasculitis. The management of this case was challenging due to the progressive and aggressive nature of the disease in a 4-year-old patient. This article aims to present the diagnostic and therapeutic strategies for the management of this condition. This report highlights a rare case of neovascular glaucoma as the first manifestation of bilateral ORV in a young child. The unusual presentation emphasizes the need for a high index of suspicion and comprehensive evaluation in cases of pediatric neovascular glaucoma. Early diagnosis and prompt, multimodal treatment are crucial in preventing irreversible vision loss in such cases.

## 1. Introduction

Neovascular glaucoma (NVG) is a secondary form of glaucoma characterized by the growth of abnormal blood vessels in the anterior segment of the eye, including the filtration angle. This neovascularization leads to angle closure, elevated intraocular pressure (IOP), and potential vision loss. NVG is most frequently associated with conditions such as central retinal vein occlusion (CRVO), diabetic retinopathy, and retinal ischemia [1,2,3,4,5] The typical clinical presentation of NVG includes ocular pain, redness, blurred vision, and neovascularization in the iris and anterior chamber angle. These changes can progress to corneal edema and significant visual impairment. However, these symptoms of NVG may be subtle or atypical in pediatric patients, often making timely diagnosis challenging [1,2,3,4,5].

The incidence of NVG in pediatric populations is relatively rare. The existing literature describes cases of NVG in children associated with conditions such as Coats’ disease, retinopathy of prematurity, central retinal vein occlusion secondary to optic nerve glioma, and medulloepithelioma [2,3,4,5]. However, the presentation of NVG as the initial manifestation of bilateral occlusive retinal vasculitis (ORV) is exceptionally uncommon, especially in children.

ORV is a condition characterized by inflammation and obstruction of the peripheral retinal blood vessels, leading to impaired blood flow to the retina. This results in retinal ischemia, which can cause complications and potentially lead to visual impairment. ORV may occur as a primary retinal condition or as a manifestation of systemic diseases, including autoimmune disorders, infections, rheumatoid diseases, or malignancies. In many cases, the etiology of ORV remains undetermined, with bilateral ORV posing a particular diagnostic challenge. If not promptly diagnosed and treated, the condition can result in potentially severe visual impairment [6,7,8]. Inflammation of these vessels is extremely rare in the pediatric population.

The management of NVG requires a multifaceted approach, including control of intraocular pressure through topical and systemic anti-glaucoma medications and treatment of underlying retinal ischemia. For cases linked to retinal vasculitis, corticosteroids and immunosuppressive therapies may be employed to reduce inflammation and prevent further damage. Intravitreal anti-vascular endothelial growth factor (anti-VEGF) injections and panretinal photocoagulation (PRP) are primary options for reducing neovascularization and improving visual outcomes [2,3,4,5].

This article aims to discuss the presentation, diagnostic challenges, and therapeutic management of NVG as the first symptom of bilateral ORV. It emphasizes the importance of maintaining a high index of suspicion in pediatric patients to prevent irreversible vision impairment. NVG associated with ORV is exceptionally rare in children and often remains underdiagnosed. Through this unique case, we aim to contribute to the understanding of this rare and complex condition while highlighting the critical need for clinician awareness in diagnosing and managing NVG in pediatric patients.

## 2. Case Report

A 4-year-old female patient presented to the Ophthalmology Department at the Children’s Memorial Health Institute in Warsaw, Poland, with symptoms of pain, redness, and epiphora in the left eye (LE). According to her parents, the symptoms had appeared suddenly a few days earlier, without any known triggers or trauma. The patient had no prior history of ophthalmic treatment, did not wear glasses, and had not experienced any previous ophthalmic diseases or surgeries. Notably, three weeks earlier she had suffered an upper respiratory tract infection accompanied by a fever of unknown etiology. Her medical history was otherwise unremarkable, with no chronic diseases, allergies, prior surgeries, or other significant health issues. The patient was born at term and vaccinated according to the current vaccination schedule. She was not taking any regular medications or dietary supplements. Furthermore, her family history was negative for ophthalmic, vascular, or oncological conditions, making her presentation particularly unusual.

The initial examination revealed a best-corrected visual acuity (BCVA) of 1.0 in the right eye (RE) and 0.1 in the LE, measured using Snellen charts. IOP measured with a Perkins applanation tonometer was significantly elevated in the LE at 35 mmHg, while the RE remained within normal limits at 18 mmHg. Biometry indicated axial lengths of 21.14 mm in the RE and 22.14 mm in the LE. Pachymetry measurements were 603 μm in the RE and 624 μm in the LE, demonstrating normal corneal thickness bilaterally. Slit-lamp examination showed ocular congestion, mild corneal edema, no purulent discharge, no inflammation in the anterior chamber and iris neovascularization (rubeosis iridis) in the LE. The anterior segment of the RE appeared normal. Fundus examination revealed peripheral retinal vascular obliterations, more pronounced in the LE than in the RE. Apart from these vascular changes on the fundus of both eyes, no other abnormalities of the optic nerve or retina were found.

**Figure 1:**
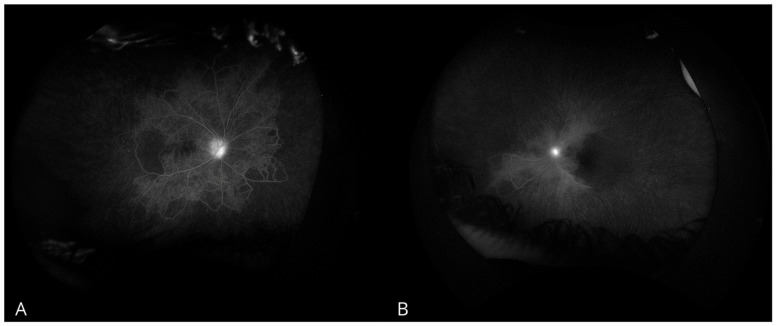
Fluorescein angiography examination. (**A**) Right eye. (**B**) Left eye.

Gonioscopic examination revealed a 360° closed angle with neovascularization in the angle of the LE, while the RE had an open angle with visible neovascularization at the trabecular meshwork. Fluorescein angiography demonstrated peripheral capillary non-perfusion with marked peripheral vascular occlusion, more severe in the LE than in the RE (Figure 1). These findings, combined with elevated IOP and neovascularization were consistent with NVG.

Given the patient’s young age, a comprehensive workup was conducted to identify potential systemic causes of retinal vasculitis. This included laboratory and imaging studies to investigate infectious etiologies, potential coexistence with systemic diseases, and malignancies. Baseline laboratory results, including complete blood count, glucose levels, electrolyte panel, C-reactive protein, creatinine, urea, and cholesterol, were normal. Coagulation tests, including homocysteine, antithrombin, protein S, protein C, factor V Leiden, factor VII, D-dimers, international normalized ratio (INR), and activated partial thromboplastin time (APTT), were also normal. Inflammatory markers, such as antinuclear antibodies (ANA), anti-neutrophil cytoplasmic antibodies (ANCA), and rheumatoid factor (RF), HLA-A29, and Anti-Ro(SSA) were negative. Tests for infectious agents, including tuberculosis, syphilis, toxoplasmosis, toxocariasis, Lyme disease, and cat scratch disease, and various viral pathogens, including human immunodeficiency virus (HIV), hepatitis B virus (HBV), hepatitis C virus (HCV), cytomegalovirus (CMV), varicella zoster virus (VZV), and Epstein–Barr virus (EBV), were unremarkable. However, serological testing was positive for herpes simplex virus type 1 (HSV-1) IgM antibodies, suggesting recent infection. Additionally, laboratory tests detected SARS-CoV-2 IgG antibodies, and human herpes virus 6 (HHV-6) IgG antibodies were detected in a viral panel. Immunological studies, including the assessment of congenital immunodeficiency and immune system disorders, such as the blastic transformation test, complement components C3 and C4, total complement activity (CH50), and levels of immunoglobulins M, A, and G, as well as an immunology consultation, revealed no abnormalities.

Imaging studies, including magnetic resonance imaging (MRI) of the brain with angiography, were normal. A chest X-ray demonstrated an enlarged heart, but no pathology was noted on the cardiac computed tomography (CT). The patient was also evaluated by specialists in pediatrics, neurology, rheumatology, gastroenterology, and genetics due to the diagnosis of retinal vasculitis.

**Figure 2:**
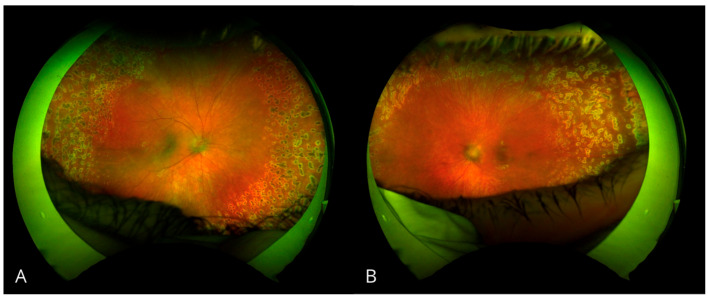
Fundus photos after panretinal laser photocoagulation. (**A**) Right eye. (**B**) Left eye.

**Figure 3:**
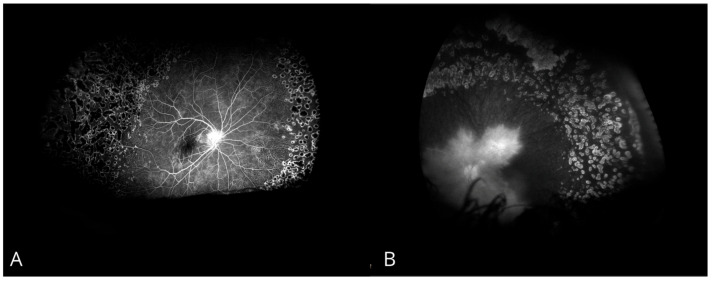
Fluorescein angiography after panretinal laser photocoagulation. (**A**) Right eye. (**B**) Left eye.

Given persistently elevated IOP in the LE, maximal anti-glaucoma therapy with topical medications was initiated. Systemic antiviral treatment was started in response to the positive HSV-1 and SARS-CoV-2 serology results. Due to vascular inflammation, steroid therapy was initiated in combination with antibiotic coverage. Due to uncontrolled IOP, the patient underwent diode laser cyclophotocoagulation in the LE. Panretinal photocoagulation of peripheral non-perfusion areas was performed twice in both eyes (Figure 2 and Figure 3).

**Figure 4:**
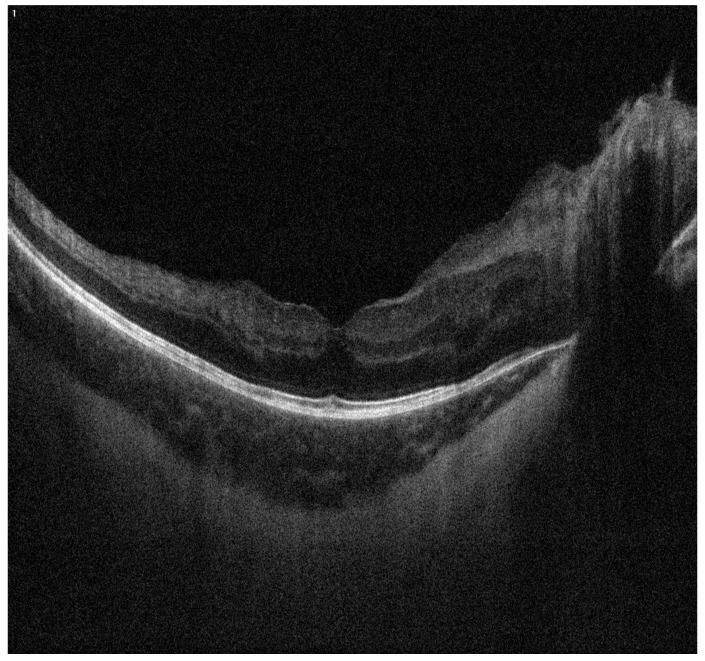
Optical coherence tomography of the retina of the right eye.

Additionally, due to iris neovascularization, an intravitreal anti-VEGF injection was administered to the LE. Despite these interventions, IOP in the LE remained elevated, necessitating a trabeculectomy with peripheral iridectomy. Following treatment, pressure normalization and stabilization of the disease were achieved. At the time optical coherence tomography of the retina was performed, disturbances in the layered architecture of the retina were noted (Figure 4).

**Figure 5:**
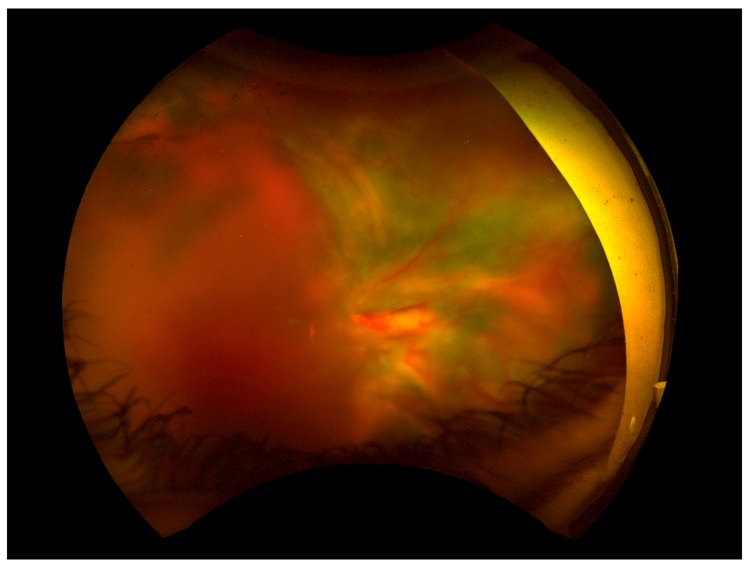
Left eye fundus image with exudative retinal detachment.

The patient was under the control of our center for one and a half years. The local condition as well as the IOP remained stable. Visual acuity stabilized at 0.8 in the RE and 0.1 in the LE. After two years, the treatment and monitoring revealed a pre-retinal hemorrhage and an exudative retinal detachment in the left eye (LE) (Figure 5).

## 3. Discussion

Neovascular glaucoma (NVG) is an uncommon complication in pediatric patients, typically arising from conditions that induce severe retinal hypoxia. This hypoxia stimulates the production of vascular endothelial growth factor (VEGF), promoting neovascularization within the eye’s anterior segment and filtration angle. NVG is most frequently associated with central retinal vein occlusion and diabetic retinopathy, although cases have been reported in children with other retinal vascular diseases. Shields et al. [3] documented a case in which a child with Coats’ disease and facioscapulohumeral dystrophy developed NVG as an initial presentation. NVG has also been observed in pediatric populations in association with conditions such as optic nerve glioma, retinal vasoproliferative tumors due to neurofibromatosis type 1, retinopathy of prematurity, and medulloepithelioma [2,3,4,5].

NVG secondary to ORV is extremely rare in children. Obstruction of the peripheral retinal blood vessels causes retinal hypoxia, which triggers increased secretion of vascular endothelial growth factor (VEGF). This stimulates intense pathological vessel proliferation, leading to neovascularization. Particularly dangerous is neovascularization in the filtration angle of the anterior chamber, which can result in angle closure. These changes can cause secondary neovascular glaucoma, ultimately leading to decreased vision, especially if left untreated.

ORV is a rare and severe inflammatory condition that primarily affects retinal blood vessels in the eyes, often causing vascular occlusion without an identifiable systemic cause. It is characterized by inflammation and obstruction of retinal vessels, leading to significant retinal ischemia. The etiology of ORV is often unclear and ambiguous. It may be associated with autoimmune and rheumatologic diseases, such as systemic lupus erythematosus and Behçet’s disease. In some instances, ORV has an infectious origin, particularly viral infections, as well as tuberculosis and syphilis. Less commonly, it manifests as a paraneoplastic syndrome linked to underlying malignancies. The differential diagnosis of ORV is broad, as retinal vasculitis may present as an isolated condition with a specific cause or as a feature of systemic disease. In cases where no underlying cause can be identified, the condition is classified as idiopathic ORV [6,7].

The differential diagnosis can often be established based on ophthalmoscopic findings, as certain fundus features are characteristic of specific etiologies, including cotton wool spots, intraretinal infiltrates, retinal necrosis, vascular necrosis, and retinal ischemia [9,10]. However, none of these features were observed in our patient, who presented solely with peripheral retinal vascular occlusion. Additionally, the patient had positive IgM titers for HSV type 1, suggesting a possible viral etiology for the vasculitis.

While a viral cause could not be definitively excluded, clinical features typically associated with HSV infection, such as frozen branch [10,11], vasculitis, and retinal necrosis, were absent during both treatment and follow-up. Despite the lack of these hallmark findings, the possibility of an atypical presentation of HSV-associated vasculitis remained, warranting the initiation of antiviral therapy.

ORV can arise from a variety of underlying causes. Cheung et al. [12] reported a case of a 13-year-old female with bilateral occlusive vasculitis associated with encephalitis caused by H1N1 influenza infection. In that case, vascular lesions were accompanied by optic disc edema and hemorrhages [12]. In contrast, our patient exhibited no additional fundus findings beyond occlusive vasculitis. The absence of other symptoms commonly linked to H1N1 infection makes an infectious etiology due to the influenza virus unlikely in this case.

Studies suggest that a wide spectrum of ocular symptoms can occur during SARS-CoV-2 infection, ranging from mild, non-threatening manifestations, such as conjunctival hyperemia, tearing, itching, and eye pain, to more severe conditions, including ORV, ischemic optic neuropathy, and inflammation of the choroid, retina, and optic nerve. Ocular manifestations are reported to be more frequent in severe cases of SARS-CoV-2 infection and occur with greater prevalence in children. According to the literature, vascular abnormalities are the most reported ocular complications following SARS-CoV-2 infection [13,14,15]. Retinal vasculitis has been described in 15 eyes, often accompanied by additional fundus findings, uveitis, and confirmed systemic diseases [13,14,15]. However, bilateral ORV directly attributable to SARS-CoV-2 infection has not been previously described [13,14,15]. Given the limited knowledge and absence of established diagnostic criteria, it cannot be ruled out that SARS-CoV-2 infection contributed to the development of ORV in our patient. The girl had an upper respiratory tract infection prior to the onset of symptoms, which occurred during the SARS-CoV-2 pandemic, but she did not have tests to confirm this etiology of infection. Although a definitive causal relationship between SARS-CoV-2 infection and ORV cannot be conclusively confirmed, it is highly plausible that the infection played a role in the development of ORV in our patient.

Recent reports on ORV have implicated drugs such as vancomycin and brolucizumab as potential triggers for ORV [16,17]. However, based on the patient’s medical history, these medications were not administered. Furthermore, genetic mutations in *CAPN5*, *TREX1*, and *TNFAIP3* have been identified as significant contributors to the development of occlusive vasculitis [17]. Unfortunately, genetic testing for these mutations was not conducted in this case. The lack of genetic testing also leaves other potential genetic factors contributing to the condition unexplored, potentially affecting the accuracy of the diagnosis. Incorporating genetic testing into future evaluations could enhance the understanding of the disease and lead to improved patient outcomes.

This case emphasizes the importance of considering atypical etiologies in pediatric NVG and highlights the diagnostic challenges posed by the absence of a clear systemic cause.

## 4. Conclusions

This case highlights a rare presentation of neovascular glaucoma as the initial manifestation of bilateral ORV in a pediatric patient. The atypical presentation of severe ocular symptoms, such as elevated intraocular pressure, corneal edema, and iris neovascularization, in such a young child is noteworthy. It underscores the importance of a thorough and systematic evaluation in cases of pediatric glaucoma. Diagnosing ORV can be challenging due to its rarity and lack of identifiable systemic causes, which often delay treatment and increase the risk of vision loss.

## Data Availability

The data presented in this study are available within the article.
